# Patient-Derived Orthotopic Xenograft Models of Pediatric Brain Tumors: In a Mature Phase or Still in Its Infancy?

**DOI:** 10.3389/fonc.2019.01418

**Published:** 2020-01-08

**Authors:** Eva Hermans, Esther Hulleman

**Affiliations:** ^1^Princess Máxima Center for Pediatric Oncology, Utrecht, Netherlands; ^2^Departments of Pediatric Oncology/Hematology, Cancer Center Amsterdam, Amsterdam UMC, Vrije Universiteit Amsterdam, Amsterdam, Netherlands

**Keywords:** PDX, pediatric, orthotopic, xenograft, WHO classification

## Abstract

In recent years, molecular profiling has led to the discovery of an increasing number of brain tumor subtypes, and associated therapeutic targets. These molecular features have been incorporated in the 2016 new World Health Organization (WHO) Classification of Tumors of the Central Nervous System (CNS), which now distinguishes tumor subgroups not only histologically, but also based on molecular characteristics. Despite an improved diagnosis of (pediatric) tumors in the CNS however, the survival of children with malignant brain tumors still is far worse than for those suffering from other types of malignancies. Therefore, new treatments need to be developed, based on subgroup-specific genetic aberrations. Here, we provide an overview of the currently available orthotopic xenograft models for pediatric brain tumor subtypes as defined by the 2016 WHO classification, to facilitate the choice of appropriate animal models for the preclinical testing of novel treatment strategies, and to provide insight into the current gaps and challenges.

## Introduction

Whilst over the past few decades there has been an improvement in the survival of patients in multiple domains within pediatric oncology, the prognosis for the majority of children with malignant brain tumors remains grim (1). Their poor survival can be attributed to a lack of efficacious therapies, and a limited understanding of the underlying genetic and biochemical abnormalities associated with this group of diseases, which has hindered the development of more effective and patient-specific treatment. In the past years, a number of recurrent mutations have been identified that allow for the identification of tumor subgroups with distinct biological characteristics (2, 3). Importantly, these molecular features have been incorporated into the new (2016) World Health Organization (WHO) classification, which now distinguishes tumor subgroups not only histologically, but also based on molecular characteristics (4). The new classification has improved the diagnosis of pediatric brain tumors, but this knowledge has not yet led to a better prognosis for pediatric brain tumor patients. In order to increase survival rates whilst decreasing treatment-related side-effects, new targeted treatments must be developed which feature subgroup-specific clinical trials, and are conducted based on the distinct underlying genetic aberrations. However, with an increasing number of tumor subgroups and consequently a decreasing number of eligible patients, it will become ever more important to test novel treatment strategies in preclinical research before proceeding to clinical trials. Representative cell lines and animal models will therefore have to be developed, representing the broad spectrum of pediatric brain tumors. To facilitate the choice of the appropriate preclinical animal model, and emphasize the need for new models that are still lacking, we here provide an overview of the currently available orthotopic xenograft models for pediatric brain tumors, divided by specific subtypes as defined by the 2016 WHO classification (4). Although multiple types of animal models are currently available for the investigation of new treatments for pediatric brain tumors *in vivo*, we will focus on patient-derived xenografts (PDXs) rather than Genetically Engineered Mouse Models (GEMMs) within which tumor-specific genetic aberrations are introduced. PDXs have been shown to have an increased reliability when reproducing the heterogeneity of the human disease, which may better reflect the therapy response in patients than GEMMs (5, 6). In addition, we will focus on the models that have been established by xenografting fresh patient-derived material rather than established human cancer cell lines that have adapted to growth under artificial culture conditions, and are generally considered less relevant for clinical translation due to a more homogeneous, undifferentiated histology (7–9). Finally, we will only consider intracranial/orthotopic models, as these models retain the tumor-host microenvironment which may play a role in tumor response (10), and tumor growth (11). Moreover, such orthotopic models closely mimic human metastasis and allow to study drug delivery past the blood-brain barrier (5, 7, 12, 13).

## PDX Models

Currently available pediatric brain tumor PDXs are established by xenografting fresh tissue, freshly isolated cell suspensions, or shortly cultured neurospheres in immunosuppressed rats (14), or immunodeficient mice (7, 15–17). Various immunocompromised mouse strains are available, with different rates of engraftment, lifespan, and sensitivity for chemotherapy or radiation (5, 9, 18). Not all strains have been fully characterized, and it is therefore essential to understand these differences when choosing the most appropriate animal model. BALB/c mice, for example, are particularly sensitive to the effects of radiation due to an unknown autosomal recessive genetic locus (19). Therefore, immunodeficient mice on a BALB/c genetic background should not be used for studies involving radiotherapy. Similarly, SCID (severe combined immunodeficient) animals are very sensitive to γ-irradiation, as they harbor a mutation in the *Prkdc* gene, which is involved in the repair of double strand DNA breaks (20). In contrast, other strains—such as Rag1-deficient (recombination activating gene 1) mice—have been reported to survive radiation doses up to 8.5 Gray, and are considered radioresistant (21). Working with mice on defined genetic backgrounds is therefore advisable for irradiation studies. The same holds true for experiments aimed at testing therapy response when DNA damaging agents are used. The response to cisplatin, doxorubicin, 5-fluoroacil, and oxaliplatin was shown to depend on PRKDC function (22), and should therefore not be tested in SCID mice. For more targeted compounds no clear guidelines exist for the choice of mouse strain, although some differences have been reported on drug sensitivity depending on drug transporters and metabolism (23). In those cases, the choice of the most appropriate PDX model should be based on the molecular subtype of the tumor.

Aside from different responses to therapy, there are also significant differences in tumor engraftment between various strains. Generally, it is believed that the level of immunodeficiency correlates with the tumor take rate (8, 9); as such, the more immunocompromised mouse strains, NOD/SCID/IL2γ-receptor null (NSG) and NOS/Rag/IL2γ-receptor null (NRG), would be most suitable strains for the implantation of primary cancerous cells, stem cells or tissue (9, 19, 24). It has been reported that these models support more robust post-engraftment tumor growth compared to double-mutant mice (25, 26), whilst maintaining the characteristics of the original primary patient tumor (27). However, studies confirming this view have only been performed with specific PDX models for hematological forms of cancer or using subcutaneous injections of tumor cells, and no convincing assessment regarding the preferred mouse strain for pediatric brain tumors has been carried out (24, 28–30).

One major limitation of the use of immunocompromised mice is that the interaction between the tumor and the immune microenvironment is partially or completely lost to ensure tumor engraftment is successful (5, 9). Consequently, the current PDX models cannot be used to study the (tumor) immune microenvironment, or to test novel immunotherapeutic treatment strategies (9). One solution to this problem has been found in the use of humanized-xenograft models (5, 9, 12, 18), in which the peripheral blood or bone marrow of the patient is co-engrafted with the tumor material into mouse strains lacking mouse natural killer cell activity (for example NSG or NRG mice) (9). Although this is a promising strategy for the testing of immunotherapy in the future, no humanized-xenograft models for pediatric brain tumors have yet been described.

Besides the choice of animal strain, other factors may influence the success rate of tumor engraftment. For instance, patient tissue can be collected either at time of diagnosis (biopsy), as part of treatment (surgical resection), or *post-mortem*. The moment of tissue collection may affect the characteristics of the PDX model, as treatment can change the molecular features of the tumor (31). As such, PDX models established from samples that are retrieved before treatment may be more suitable to test new therapies that can be implemented in the initial treatment schedules, while PDX models from autopsy samples, representing the late stage of disease, may be more appropriate to study resistance mechanisms and treatment effects (32).

In addition, various methods are used for the processing of the tumor cells before injection. Although occasionally whole tumor pieces have been used for implantation (33, 34), the most used method to establish pediatric brain tumor PDX models, is the preparation of cell suspensions either by dissociation of neurospheres or directly from surgical specimen (Table A1). Alternatively, tumor cells can be enriched for brain tumor-initiating cells (BTICs) by sorting for CD133+ cells (35), grown as an adherent layer (31, 36–44), transplanted in the thalamus or subcutaneously to expand the tumor cells (32, 40, 45, 46), or injected intracranially after serial transplantation (16, 35, 40, 46–55).

Although subcutaneous propagation has been shown to retain tumor characteristics and to decrease the time required for the PDX model procedure (7), no significant differences appear to exist between the direct- and indirect xenografting of tumor cells. In a head to head comparison of tumor models, generated by the injection of tumor cells derived directly from the patient and implantation of cultured cells, no variance was observed in tumorigenicity or histopathology of the xenograft (32). The authors did however find a discrepancy in survival time, with xenograft models obtained from cells in culture living longer (see Table A1), correlating to a greater degree with patient survival. This discrepancy between the direct- and indirect method could originate from inequivalent numbers of injected tumor cells, or the presence of stroma and microenvironment in direct implantation.

Besides a better correlation with patient survival, indirect xenografting, encompassing a cell culture step before intracranial implantation, additionally allows for the introduction of the *Firefly luciferase* gene by lentiviral transduction, facilitating non-invasive monitoring of tumor growth by bioluminescent imaging (BLI) in preclinical therapeutic studies (56). Although a temporary culture step as an adherent monolayer may be needed for effective transduction (57), cells are generally grown as neurospheres, since spheroid cultures have been shown to have a greater degree of genetic stability compared to cells grown in attachment (58). Independent of the culture conditions or method of implantation, PDXs should always be compared to the original tumor to validate the models. Preferably this is done both histologically and by molecular analyses, e.g., by confirmation of copy number variations/tumor-specific mutations or DNA methylation profiling. Such validation is extremely important, as some studies even suggest that the presence of stroma cells in *post-mortem* tissue may generate murine tumors rather than human xenografts (59, 60).

The large variety of available methods and mouse strains indicates that, until recently, no clear consensus existed in the field regarding the best model set-up. However, in the past decade multiple consortia have been founded, such as the Pediatric Preclinical Testing Consortium, the Childhood Solid Tumor Network, the Children's Oncology Group (COG), and the European EurOPDX resource, that collect and validate PDX models to increase the reproducibility of PDX studies (16). Although currently only few pediatric PDX models are included in the abovementioned databases, these initiatives emphasize the importance of a validated set-up. Furthermore, in order to assure the quality of newly established PDX models, a PDX models Minimal Information standard (PDX-MI) has been developed that defines the minimal information regarding the clinical characteristics and the procedures of implantation in a host mouse strain (31). For all these models it will be important to validate to which extent the xenograft tumor diverges from the donor tumor, both molecularly and histologically (8). However, the provision of such data, as well as peruse of the clinical patient information, might be challenging due to patient privacy or data inaccessibility (31).

## Future Perspectives

Whilst the number of available orthotopic xenograft models for pediatric brain tumor research is growing, some tumor types are still underrepresented. Models for craniopharyngioma, germinoma, embryonal tumors with multilayered rosettes (ETMR), pineoblastoma, diffuse astrocytoma, oligodendroglioma, and cancers belonging to the “other astrocytic tumors/gliomas” are scarce, and no models have currently been described for e.g., choroid plexus tumors. This paucity may be attributed to a minimal research interest into certain tumor types, the limited availability of tumor material, or a low tumor take-rate (17). Failure of tumor engraftment often occurs with the less aggressively growing (low-grade) tumors, such as pilocytic astrocytoma (61). For some of these tumor types, the use of more invading cells from a metastatic site (62), or samples from recurrent tumors might be an interesting alternative, as more aggressive tumor cells are thought to have a higher take rate *in vivo* (18). Care however needs to be taken to assure the practical use of such models, as recurrences and metastatic clones may differ from the primary tumor at diagnosis. Alternatively, more effective tumor-specific protocols may have to be developed. So far, only few comparative studies have been performed to determine the most optimal protocols per tumor type, with regard to sample size, sample processing, and mouse strain (17). In addition, the choice of animal model and experimental set-up may vary, depending on the research question; for low-grade tumors, for example, studies may be aimed at diminishing treatment-related side-effects, while survival studies will be more relevant for tumor subtypes with a poor prognosis.

Whilst appropriate PDX models for some tumor types are still missing, other pediatric brain tumor types seem to be more strongly represented. This especially holds true for models of glioblastoma, diffuse midline glioma, ependymoma, and medulloblastoma. Preclinical research in these fields is expanding, partly due to the raised interest in these tumor types, and to the increased availability of tumor material. For example, the development of autopsy protocols and the reintroduction of surgical biopsies for diffuse midline gliomas (63) has boosted preclinical research for these tumors, leading to the development of several animal models (16). Yet, more PDX models may be required for these tumor types as well, to cover different subgroups, stages, and heterogeneity of the disease. Full tumor dynamics may be captured by the collection of paired tumor samples at the time of diagnosis and at autopsy, while intratumoral heterogeneity may be covered by the sampling of multiple lesions from the same tumor in rapid autopsy protocols (64). Additional PDX models comprising the complete spectrum of the disease are needed to confirm the reproducibility of preclinical results, and to ensure clinical relevance of laboratory findings.

Despite the presence of a relatively high number of pediatric glioma models, PDXs covering *IDH1* mutations are lacking. Moreover, many described PDX models for pediatric glioma have not been molecularly characterized (16, 35, 38, 48, 65), even though mutation analysis could classify them as belonging to specific biological subgroups (66). The same holds true for ependymoma (14, 38, 45, 46, 67) and, to a lesser extent, medulloblastoma models (38, 42–44, 68). For other tumor types, such as pineoblastoma, or germ cell tumors no molecular subgroups have yet been identified. Proper model validation and characterization of the available PDXs will be essential to test new therapies, especially when targeted therapy is applied. Many of the currently available PDX models without molecular designation have been established in the early 2000s, and these models may still be useful, provided that molecular profiling is performed. This might be an option for tumor types for which less PDXs are currently available, such as the atypical teratoid rhabdoid tumors (AT/RTs), a relatively rare, but highly aggressive pediatric brain tumor with a poor survival (69), which would benefit from preclinical *in vivo* studies to ameliorate prognosis and diminish long-term sequelae. One should however keep in mind that validation of those models by comparing the molecular features of the PDX with the original tumor will often not be possible. In such cases, models may be validated by comparing RNAseq-, whole genome sequencing-, and DNA methylation profiles with cohorts of patient data to ensure their representability of the human disease.

In order to translate preclinical findings to the clinic, the proper choice of animal model and experimental set-up will be paramount. Improved PDX models may be used for personalized medicine purposes, where the predictive value of therapy for a certain patient is determined based on a personal panel of mouse tumors. However, such a personalized approach is currently hampered by the time that is needed to develop these models, costs, and the variable rate of engraftment. Alternatively, multiple tumor-specific animal models may be used to conduct so-called Mouse Clinical Trials (MCTs). MCTs use small numbers of mice per treatment arm across a large number of PDX models, resembling human clinical trials more closely than preclinical trials in which large numbers of a specific PDX model are used (70). MCTs will help researchers to understand the correlation of specific genetic factors to therapy response, and may allow to predict patient response, as well as correct patient stratification. For this reason, additional, fully characterized models need to be developed with a special focus on the poorly represented subtypes. These models may be used to determine the best therapeutic regimes for each tumor subtype to implement in standard protocols.

In summary, although progress has been made in the development of orthotopic xenograft models for pediatric brain tumors, there is a clear imbalance in the number of PDX models for different tumor types, and a high variability in methodology and animal strains used. Combined efforts of neurosurgeons, pathologists, pediatric oncologists and preclinical researchers will be needed to develop additional animal models for the design of effective therapeutic strategies.

## Author Contributions

EHe wrote the first draft of the manuscript, while EHu revised the manuscript. Both authors contributed to the conception, design, and approved the submitted version.

### Conflict of Interest

The authors declare that the research was conducted in the absence of any commercial or financial relationships that could be construed as a potential conflict of interest.

## Appendix

**Table A1:** Overview of available orthotopic xenograft models per tumor entity, based on the 2016 WHO classification of tumors of the central nervous system.

**Model name**	**Tumor classification**	**Tumor location**	**Molecular classification**	**Moment of tumor collection**	**Age in years (y) or months (mo) and sex donor **♀♂****	**Mouse strain and age**	**Tumor preparation before injection**	**Injection site**	**BLI**	**Time to tumor growth/euthanasia**	**Source**	**References**
**DIFFUSE ASTROCYTIC AND OLIGODENDROGLIAL TUMORS**												
bGB1	Giant cell glioblastoma	Cerebrum (frontal lobe)	ND	Surgical resection	3.6 y	ND	Short-term adherent cell culture	Right cerebral hemisphere (ML +2 mm, AP +2 mm)	–	ND	University of Birmingham	(38)
CCHMC-DIPG-1	Diffuse midline glioma, H3K27M mutant	ND	H3.3K27M	ND	ND	NSG, postnatal day 2	Short-term cell culture in spheroids	4th ventricle (AP −3 mm, DV −3 mm)	+	16–19 days	On request (Dr. Drissi, Cincinnati Children's Hospital)	(71)
DIPG-PBTR3	Diffuse midline glioma, H3K27M mutant	Ventral pons	H3.3K27M	Autopsy	5 y ♂	NSG, postnatal day 2	Short-term cell culture in spheroids	4th ventricle (AP −3 mm, DV −3 mm)	+	6 months to clinical symptoms	On request	(71)
GBM-311FH	Glioblastoma, IDH wild-type	Cortex (left temporal lobe)	Hypermutator	Surgical resection	10.8 y ♂	NSG	Cell suspension from surgical specimen	Right cerebral hemisphere (ML +1 mm, AP +1.5 mm, DV −2 mm)	–	77–85 days	BTRL (Brain Tumor Resource Lab—https://research.fhcrc.org)	(72)
GBM-611FH	Glioblastoma, IDH wild-type	Cortex (left temporal lobe)	Hypermutator	Autopsy (recurrence)	11.3 y ♂	NSG	Cell suspension from surgical specimen	Right cerebral hemisphere (ML +1 mm, AP +1.5 mm, DV −2 mm)	–	79–128 days	BTRL (Brain Tumor Resource Lab—https://research.fhcrc.org)	(72)
GU-pBT-7	Diffuse midline glioma, H3K27M mutant	Right hemisphere (thalamus)	H3.1K27M, EGFR/KRAS amplification, CCND deletion	Surgical resection (primary tumor)	4.2 y ♂	NOD/SCID, 6–8 weeks	Short-term adherent cell culture	Frontal cortex ML +2 mm, AP +1 mm, DV −2.5 mm	–	120–125 days	On request	(37)
GU-pBT-10	Glioblastoma NOS	Right hemisphere (relapse)	CDKN2A/B deletion	Surgical resection (recurrence)	10.4 y ♂	NOD/SCID, 6–8 weeks	Short-term adherent cell culture	Frontal cortex ML +2 mm, AP +1 mm, DV −2.5 mm	–	215–330 days	On request	(36)
GU-pBT-15	Diffuse midline glioma, H3K27M mutant	Brain stem	H3.3K27M	Surgical resection (primary tumor)	12.5 y ♀	NOD/SCID, 6–8 weeks	Short-term adherent cell culture	Frontal cortex ML +2 mm, AP +1 mm, DV −2.5 mm	–	310–400 days	On request	(36)
GU-pBT-19	Diffuse midline glioma, H3K27M mutant	Right hemisphere (thalamus)	H3.3K27M, RB deletion	Surgical resection (primary tumor)	6.2 y ♂	NOD/SCID, 6–8 weeks	Short-term adherent cell culture	Frontal cortex ML +2 mm, AP +1 mm, DV −2.5 mm	–	285–350 days	On request	(36)
GU-pBT-23	Glioblastoma NOS	Left hemisphere (temporal)	PDGFRA/ CDK4/MDM2 amplification	Surgical resection (primary tumor)	2.9 y ♀	NOD/SCID, 6–8 weeks	Short-term adherent cell culture	Frontal cortex ML +2 mm, AP +1 mm, DV −2.5 mm	–	70–75 days	On request	(37)
GU-pBT-28	Glioblastoma NOS	Pons (cerebellopontine angle)	EGFR amplification, NF1/ CDKN2A/B deletion	Surgical resection (primary tumor)	11.1 y ♀	NOD/SCID, 6–8 weeks	Short-term adherent cell culture	Frontal cortex ML +2 mm, AP +1 mm, DV −2.5 mm	–	130–155 days	On request	(37)
HSJD-DIPG-07	Diffuse midline glioma, H3K27M mutant	Pons	H3.3K27M, ACVR1 R206H	Autopsy	9.9 y ♂	Athymic nude Foxn1nu, 6 weeks	Short-term cell culture in spheroids	Pons (ML +1 mm, AP −0.8 mm, DV −4.5 mm)	+	38–74 days	On request (Dr. Montero-Carcaboso, Barcelona)	(73)
Ibs-W0128DIPG/ Li-F	Glioblastoma, IDH wild-type	Pons	H3 WT, ACVR1 G328V, PIK3CA Q546K	Autopsy	8.5 y ♂	NOD/SCID	Cell suspension from surgical specimen	Pons (DV −5.2 mm)	–	37–70 days	On request (Dr. Li, Houston)	(47)
IC-1128GBM	Glioblastoma NOS	Cerebrum	ND	Surgical resection (recurrence)	8.6 y ♂	Rag2/SCID, 6–8 weeks	Cell suspension from surgical specimen	Right cerebral hemisphere (ML +1 mm, AP +1.5 mm, DV −3 mm)	–	150–180 days	On request	(16)
IC-1406 GBM	Glioblastoma NOS	Cerebrum	ND	Surgical resection	5 y ♀	Rag2/SCID, 6–8 weeks	Cell suspension from surgical specimen	Right cerebral hemisphere (ML +1 mm, AP +1.5 mm, DV −3 mm)	–	67–79 days	On request	(48)
IC-1502 GBM	Giant cell glioblastoma	Cerebrum	ND	Surgical resection	4.6 y ♀	Rag2/SCID, 6–8 weeks	Cell suspension from surgical specimen	Right cerebral hemisphere (ML +1 mm, AP +1.5 mm, DV −3 mm)	–	77–96 days	On request	(48)
IC-1621 GBM	Glioblastoma NOS	Cerebrum	ND	Surgical resection	6 y ♂	Rag2/SCID, 6–8 weeks	Cell suspension from surgical specimen	Right cerebral hemisphere (ML +1 mm, AP +1.5 mm, DV −3 mm)	–	125–160 days	On request	(48)
IC-2305 GBM	Glioblastoma NOS	Cerebrum	ND	Surgical resection	9 y ♀	Rag2/SCID, 6–8 weeks	Cell suspension from surgical specimen	Right cerebral hemisphere (ML +1 mm, AP +1.5 mm, DV −3 mm)	–	ND	On request	(48)
IC-3704 GBM	Glioblastoma NOS	Cerebrum	ND	Surgical resection	12 y ♂	Rag2/SCID, 6–8 weeks	Cell suspension from surgical specimen	Right cerebral hemisphere (ML +1 mm, AP +1.5 mm, DV −3 mm)	–	ND	On request	(35)
IC-3752 GBM	Glioblastoma NOS	Left hemisphere (frontal)	H3 WT	Surgical resection (recurrence)	4 y ♀	Rag2/SCID, 6–8 weeks	Cell suspension from surgical specimen	Right cerebral hemisphere (ML +1 mm, AP +1.5 mm, DV −3 mm)	–	ND	On request	(35)
IC-4687GBM	Glioblastoma NOS	Right hemisphere (thalamus)	H3 WT	Surgical resection (at diagnosis)	7 y ♂	NOD/SCID, 6–8 weeks	Cell suspension from surgical specimen	Right cerebral hemisphere (ML +1 mm, AP +1.5 mm, DV −3 mm)	–	40–117 days	On request	(74)
IC-R0315GBM	Glioblastoma NOS	Left hemisphere (parietal)	H3 WT	Autopsy	9 y ♀	NOD/SCID, 6–8 weeks	Cell suspension from surgical specimen	Right cerebral hemisphere (ML +1 mm, AP +1.5 mm, DV −3 mm)	–	35–47 days	On request	(74)
ICb-1227AA	Anaplastic astrocytoma NOS (secondary)	Cerebellum	ND	Surgical resection	16.9 y ♀	Rag2/SCID, 6–8 weeks	Cell suspension from surgical specimen	Cerebellum (ML +1 mm, AP −1 mm, DV −3 mm)	–	62–80 days	On request	(16)
JHH-DIPG-01	Diffuse midline glioma, H3K27M mutant	Pons	H3.3K27M	Autopsy	8 y ♂	Athymic nu/nu	Short-term cell culture in spheroids	Brainstem (ML +1 mm, AP −5 mm, DV −3.5 mm)	–	230–245 days	On request	(75)
NEM273	Diffuse midline glioma, H3K27M mutant	Pons	H3.1K27M, ACVR1 G328E	Biopsy	4.6 y ♂	Athymic nude, 4–6 weeks	Short-term adherent cell culture	Pons (ML +1 mm, AP −1 mm, DV −5 mm)	+	220–258 days	On request	(32)
NEM285	Diffuse midline glioma, H3K27M mutant	Pons	H3.3K27M, TP53 A159V	Biopsy	7.1 y ♂	Athymic nude, 4–6 weeks	Short-term adherent cell culture	Pons (ML +1 mm, AP −1 mm, DV −5 mm)	+	174–224 days	On request	(32)
							Cell suspension from surgical specimen	Thalamus/Pons (ML +2 mm, AP −3 mm, DV −3.5 mm/ML +1 mm, AP −1 mm, DV −5 mm)	–	117–129 days	On request	
NEM289	Diffuse midline glioma, H3K27M mutant	Pons	H3.2K27M, TP53 W146*	Biopsy	4.7 y ♂	Athymic nude, 4–6 weeks	Short-term adherent cell culture	Pons (ML +1 mm, AP −1 mm, DV −5 mm)	+	228–270 days	On request	(32)
							Cell suspension from surgical specimen	Thalamus/Pons (ML +2 mm, AP −3 mm, DV −3.5 mm/ML +1 mm, AP −1 mm, DV −5 mm)	–	93–111 days	On request	
NEM290	Diffuse midline glioma, H3K27M mutant	Pons	H3.3K27M, TP53 R175H	Biopsy	11.6 y ♀	Athymic nude, 4–6 weeks	Short-term adherent cell culture	Pons (ML +1 mm, AP −1 mm, DV −5 mm)	+	131–139 days	On request	(32)
							Cell suspension from surgical specimen	Thalamus/Pons (ML +2 mm, AP −3 mm, DV −3.5 mm/ML +1 mm, AP −1 mm, DV −5 mm)	–	68–92 days	On request	
NEM292	Diffuse midline glioma, H3K27M mutant	Pons	H3.3K27M, TP53 P151T	Biopsy	5.2 y ♀	Athymic nude, 4–6 weeks	Short-term adherent cell culture	Pons (ML +1 mm, AP −1 mm, DV −5 mm)	+	61–73 days	On request	(32)
NEM325	Diffuse midline glioma, H3K27M mutant	Pons	H3.3K27M	Biopsy	5.5 y ♀	Athymic nude, 4–6 weeks	Cell suspension from surgical specimen	Thalamus/Pons (ML +2 mm, AP −3 mm, DV −3.5 mm/ML +1 mm, AP −1 mm, DV −5 mm)	–	87–111 days	On request	(32)
NEM328	Diffuse midline glioma, H3K27M mutant	Pons	H3.1K27M, ACVR1 G328V	Biopsy	3.5 y ♀	Athymic nude, 4–6 weeks	Short-term adherent cell culture	Pons (ML +1 mm, AP −1 mm, DV −5 mm)	+	239–295 days	On request	(32)
							Cell suspension from surgical specimen	Thalamus/Pons (ML +2 mm, AP −3 mm, DV −3.5 mm/ML +1 mm, AP −1 mm, DV −5 mm)	–	147–211 days	On request	
NEM335	Diffuse midline glioma, H3K27M mutant	Pons	H3.3K27M, TP53 R248Q	Biopsy	6.2 y ♂	Athymic nude, 4–6 weeks	Cell suspension from surgical specimen	Thalamus/Pons (ML +2 mm, AP −3 mm, DV −3.5 mm/ML +1 mm, AP −1 mm, DV −5 mm)	–	126–134 days	On request	(32)
NEM347	Diffuse midline glioma, H3K27M mutant	Pons	H3.3K27M, TP53 R273C	Biopsy	9.1 y ♂	Athymic nude, 4–6 weeks	Cell suspension from surgical specimen	Thalamus/Pons (ML +2 mm, AP −3 mm, DV −3.5 mm/ML +1 mm, AP −1 mm, DV −5 mm)	–	117–125 days	On request	(32)
NEM353	Diffuse midline glioma, H3K27M mutant	Pons	H3.3K27M	Biopsy	6.5 y ♀	Athymic nude, 4–6 weeks	Cell suspension from surgical specimen	Thalamus/Pons (ML +2 mm, AP −3 mm, DV −3.5 mm/ML +1 mm, AP −1 mm, DV −5 mm)	–	81 days	On request	(32)
nOLIG1	Oligodendroglioma NOS	Cerebrum (right fronto temporo-parietal)	ND	Surgical resection	6.5 y	ND	Short-term adherent cell culture	Right cerebral hemisphere (ML +2 mm, AP +2 mm)	–	ND	Children's Brain Tumour Research Centre, Nottingham	(38)
PBT-01FH	Diffuse midline glioma, H3K27M mutant	Cortex, bilateral thalamic	H3.1K27M	Autopsy (recurrence)	5 y ♀	NSG	Cell suspension from surgical specimen	Right cerebral hemisphere (ML +1 mm, AP +1.5 mm, DV −2 mm)	–	89–116 days	BTRL (Brain Tumor Resource Lab—https://research.fhcrc.org)	(72)
PBT-02FH	Anaplastic astrocytoma, NOS	Cortex	CDK4 amplification, FGFR1 mutation	Autopsy (recurrence)	14.8 y ♂	NSG	Cell suspension from surgical specimen	Right cerebral hemisphere (ML +1 mm, AP +1.5 mm, DV −2 mm)	–	52–121 days	BTRL (Brain Tumor Resource Lab—https://research.fhcrc.org)	(72)
PBT-05FH	Glioblastoma, IDH wild-type	Cortex, right frontal	Myc amplification	Surgical resection (recurrence)	9.1 y ♀	NSG	Cell suspension from surgical specimen	Right cerebral hemisphere (ML +1 mm, AP +1.5 mm, DV −2 mm)	–	37–42 days	BTRL (Brain Tumor Resource Lab—https://research.fhcrc.org)	(72)
PBT-06FH	Glioblastoma, IDH wild-type	Cortex, right frontoparietal	p 53 mutation, CDK4 amplification	Autopsy (recurrence)	15.9 y ♀	NSG	Cell suspension from surgical specimen	Right cerebral hemisphere (ML +1 mm, AP +1.5 mm, DV −2 mm)	–	131–326 days	BTRL (Brain Tumor Resource Lab—https://research.fhcrc.org)	(72)
QCTB-R059	Diffuse midline glioma, H3K27M mutant	Thalamus	H3.3K27M	Surgical resection	10.4 y ♀	NSG, postnatal day 35	Short-term cell culture in spheroids	Thalamus (ML +0.8 mm, AP −1 mm, DV −3.5 mm)	+	12–14 days	Queensland Children's Medical Research Institute, Brisbane	(76)
SF7761	Diffuse midline glioma, H3K27M mutant	Pons	H3.3K27M (hTERT modified)	Biopsy	6 y ♀	Athymic nu/nu, 6 weeks	Short-term cell culture in spheroids	Pontine tegmentum (ML +1.5 mm, DV −5 mm)	+	106–130 days	On request	(77)
SF8628	Diffuse midline glioma, H3K27M mutant	Pons	H3.3K27M, p53 mutation	Biopsy	3 y ♀	Athymic nu/nu, 5 weeks	Short-term adherent cell culture	Pontine tegmentum (ML +1.5 mm, DV −5 mm)	+	66–70 days	On request	(39)
SU-pcGBM1	Glioblastoma NOS	Cortex	ND	ND	ND	NOD/SCID, 6–8 weeks	Short-term cell culture in spheroids	Left hemisphere (ML−2 mm, AP −2 mm, DV −3.5 mm)	+	ND	On request (Dr. Monje, Stanford)	(65)
SU-pcGBM2	Glioblastoma, IDH wild-type	Frontal lobe	P53 mutation, EGFR amplification	Biopsy	15 y ♂	NSG, postnatal day 35	Short-term cell culture in spheroids	Right hemisphere (ML +0.5 mm, AP +1 mm, DV −1.75 mm)	+	126–163 days	On request	(11)
SU-DIPG-I	Anaplastic astrocytoma, IDH wiltd-type	Pons	H3 WT, p53 mutation	Autopsy	5 y ♂	NSG, postnatal day 2	Short-term cell culture in spheroids	4th ventricle/lateral ventricles (ML +1 mm, AP −3 mm, DV −3 mm/ML +1 mm, AP +2 mm, DV −2 mm	–	26 weeks to clinical symptoms	On request (Dr. Monje, Stanford)	(78)
SU-DIPG-VI	Diffuse midline glioma, H3K27M mutant	Pons	H3.3K27M, p53 mutation	Autopsy	7 y ♀	NSG, postnatal day 2	Short-term cell culture in spheroids	4th ventricle/pons (AP −3 mm, DV −3 mm)	+	≤2 months (BLI)	On request	(47)
SU-DIPG-XIIIP*	Diffuse midline glioma, H3K27M mutant	Pons	H3.3K27M	Autopsy	6 y ♀	NSG, postnatal day 43	Short-term cell culture in spheroids	4th ventricle/pons (ML +0.8 mm, AP −0.5 mm, DV −5 mm)	+	19–28 days	On request	(79)
SU-DIPG-XIIIFL	Diffuse midline glioma, H3K27M mutant	Frontal lobe metastasis	H3.3K27M	Autopsy	6 y ♀	NSG, postnatal day 2	Short-term cell culture in spheroids	4th ventricle/pons (ML +0.8 mm, AP −0.5 mm, DV −5 mm)	+	ND	On request	(79)
SU-DIPG-XIX	Diffuse midline glioma, H3K27M mutant	Pons	H3.3K27M	Autopsy	2 y ♂	NSG, postnatal day 35	Short-term cell culture in spheroids	Pons (ML +1 mm, AP −0.8 mm, DV −5 mm)	+	ND	On request	(80)
SU-pSCG-1	Diffuse midline glioma, H3K27M mutant	spinal cord	H3.3K27M	Autopsy	12 y ♂	NSG, postnatal day 35	Short-term cell culture in spheroids	Medulla (ML +0.7 mm, AP −3.5 mm, DV −4.5 mm)	+	ND	On request (Dr. Monje, Stanford)	(76)
TT10603	Diffuse midline glioma, H3K27M mutant	Pons	H3.3K27M, TP53 R141C	Surgical resection	7 y ♂	NSG	Short-term adherent cell culture	Brainstem (ML +1 mm, AP −1.5 mm, DV −4.5 mm)	–	172 days to onset (MRI)	On request	(40)
TT10630	Diffuse midline glioma, H3K27M mutant	Pons	H3.3K27M, PPM1D S516X	Biopsy	4 y ♀	NSG	Short-term adherent cell culture	Brainstem (ML +1 mm, AP −1.5 mm, DV −4.5 mm)	–	186 days to onset (MRI)	On request	(40)
TT10714	Diffuse midline glioma, H3K27M mutant	Pons	H3.3K27M, PPM1D C478X	Surgical resection	6 y ♀	NSG	Short-term adherent cell culture	Brainstem (ML +1 mm, AP −1.5 mm, DV −4.5 mm)	–	155 days to onset (MRI)	On request	(40)
VUMC-DIPG-F	Diffuse midline glioma, H3K27M mutant	Pons	H3.3K27M	Biopsy	7 y ♂	FVB athymic, 6–8 weeks	Short-term cell culture in spheroids	Pons (ML +0.8 mm, AP −1 mm, DV −4.5 mm)	+	120–179 days	On request	(81)
**OTHER ASTROCYTIC TUMORS**												
IC-3635 PXA	Pleomorphic xanthoastrocytoma (grade II)	Left temporal lobe	BRAF V600E, CDKN2A deletion	Surgical resection	10 y ♀	NOD/SCID, 6–8 weeks	Cell suspension from surgical specimen	Right cerebral hemisphere (ML +1 mm, AP +1.5 mm, DV −3 mm)	–	175–255 days	On request	(82)
**EPENDYMAL TUMORS**												
BT-44	Anaplastic ependymoma	Posterior fossa	ND	ND	2 y ♀	Athymic nu/nu, 5–6 weeks	Cell suspension from surgical specimen	Caudate nucleus	–	100–155 days	On request	(46)
BT-57	Anaplastic ependymoma	Posterior fossa (focal)	ND	ND	10 mo ♂	Athymic nu/nu, 5–6 weeks	Cell suspension from surgical specimen	Caudate nucleus	–	100–155 days	On request	(46)
D528 EP-X	Ependymoma	Posterior fossa	ND	Biopsy	2.5 y ♀	BALB/c nu/nu, 3–4 weeks	Cell suspension from surgical specimen	Right cerebral hemisphere	–	± 85 days	On request	(67)
D612 EP-X	Ependymoma	Posterior fossa	ND	Biopsy	1.1 y ♀	BALB/c nu/nu, 3–4 weeks	Cell suspension from surgical specimen	Right cerebral hemisphere	–	± 72.5 days	On request	(67)
E520-PF1	Ependymoma	Infratentorial	A/CIMP (+)	Surgical resection	ND	NSG 8–12 weeks	Short-term adherent cell culture	Right cerebral hemisphere (ML +1 mm, AP +1.5 mm, DV −3 mm)	+	30–59 days	On request	(41)
EPD-210FH	Anaplastic ependymoma	Posterior fossa	PFA	Autopsy (recurrence)	10 y ♂	NSG	Cell suspension from surgical specimen	Right cerebral hemisphere (ML +1 mm, AP +1.5 mm, DV −2 mm)	–	75–103 days	BTRL (Brain Tumor Resource Lab—https://research.fhcrc.org)	(72)
EPD-613FH	Ependymoma, RELA fusion positive (grade III)	ND	RELA	Surgical resection (recurrence)	16 y ♂	NSG	Cell suspension from surgical specimen	Right cerebral hemisphere (ML +1 mm, AP +1.5 mm, DV −2 mm)	–	137–223 days	BTRL (Brain Tumor Resource Lab—https://research.fhcrc.org)	(72)
EPD-710FH	Anaplastic ependymoma	Posterior fossa	PFA	Surgical resection	2.8 y ♂	NSG	Cell suspension from surgical specimen	Right cerebral hemisphere (ML +1 mm, AP +1.5 mm, DV −2 mm)	–	115–326 days	BTRL (Brain Tumor Resource Lab—https://research.fhcrc.org)	(72)
EPN1	Anaplastic ependymoma	Posterior fossa	ND	Surgical resection	ND	Wistar Rat, treated with immunosuppressant drugs	Short-term adherent cell culture	3rd ventricle (ML+1.4 mm, AP +0.8 mm, DV −3.8 mm)	FL	≤45 days (X-ray/fluorescent imaging)	On request	(14)
EPN2	Anaplastic ependymoma	Posterior fossa	ND	Surgical resection	ND	Wistar Rat, treated with immunosuppressant drugs	Short-term adherent cell culture	3rd ventricle (ML+1.4 mm, AP +0.8 mm, DV −3.8 mm)	FL	≤45 days (X-ray/fluorescent imaging)	On request	(14)
EPN3	Anaplastic ependymoma	Posterior fossa	ND	Surgical resection	ND	Wistar Rat, treated with immunosuppressant drugs	Short-term adherent cell culture	3rd ventricle (ML+1.4 mm, AP +0.8 mm, DV −3.8 mm)	FL	≤45 days (X-ray/fluorescent imaging)	On request	(14)
EPN4	Anaplastic ependymoma	Posterior fossa	ND	Surgical resection	ND	Wistar Rat, treated with immunosuppressant drugs	Short-term adherent cell culture	3rd ventricle (ML+1.4 mm, AP +0.8 mm, DV −3.8 mm)	FL	≤45 days (X-ray/fluorescent imaging)	On request	(14)
EPN5	Anaplastic ependymoma	Posterior fossa	ND	Surgical resection	ND	Wistar Rat, treated with immunosuppressant drugs	Short-term adherent cell culture	3rd ventricle (ML+1.4 mm, AP +0.8 mm, DV −3.8 mm)	FL	≤45 days (X-ray/fluorescent imaging)	On request	(14)
EPP	Ependymoma	4th ventricle	SEC61G-EGFR gene fusion (subclone)	Surgical resection (recurrence)	3.2 y ♂	CD1 nu/nu, 5 weeks	Short-term cell culture in spheroids	4th ventricle (ML +0.2 mm, AP −6 mm, DV −4 mm)	–	70–104 days	On request	(45)
EPV	Ependymoma	Posterior fossa	ND	Surgical resection (recurrence)	1.9 y ♂	CD1 nu/nu, 5 weeks	Short-term cell culture in spheroids	4th ventricle (ML +0.2 mm, AP −6 mm, DV −4 mm)	–	68–149 days	On request	(45)
IC-1425EPN	Ependymoma, RELA fusion positive (grade III)	supratentorial	C11orf95-RELA fusion	Surgical resection (recurrence)	9 y ♂	Rag2/SCID, 6–8 weeks	Cell suspension from surgical specimen	Right cerebral hemisphere (ML +1 mm, AP +1.5 mm, DV −3 mm)	–	85–180 days	On request	(50)
nEPN1	Ependymoma RELA fusion positive (grade II)	supratentorial (right parietal)	C11orf95-RELA fusion	Surgical resection (recurrence)	13.5 y ♂	ND	Short-term adherent cell culture	Right cerebral hemisphere (ML +2 mm, AP +2 mm)	–	ND	Children's Brain Tumour Research Centre, Nottingham	(38)
nEPN2	Ependymoma	4th ventricle	ND	Surgical resection	3.4 y	ND	Short-term adherent cell culture	Right cerebral hemisphere (ML +2 mm, AP +2 mm)	–	ND	Children's Brain Tumour Research Centre, Nottingham	(38)
**TUMORS OF THE PINEAL REGION**												
PBT-08FH	Pineoblastoma	Pineal region	Drosha (splice site and splice site mutation)	Surgical resection	11.2 y ♀	NSG	Cell suspension from surgical specimen	Right cerebellum (ML +2 mm, AP −2 mm, DV −2 mm)	–	245 days	BTRL (Brain Tumor Resource Lab—https://research.fhcrc.org)	(72)
Pineo-113FH	Pineoblastoma	ND	ND	Surgical resection	8 y ♂	NSG	Cell suspension from surgical specimen	Right cerebral hemisphere (ML +1 mm, AP +1.5 mm, DV −2 mm)	–	162–301 days	BTRL (Brain Tumor Resource Lab—https://research.fhcrc.org)	(72)
**EMBRYONAL TUMORS—MEDULLOBLASTOMA**												
BO-101	Medulloblastoma, NOS	Cerebellum	ND	Surgical resection	9 y ♂	Athymic nu/nu, 3–4 weeks	Short-term adherent cell culture	Right cerebral hemisphere	–	ND	On request	(42)
CHLA-01-MED = CRL-3021	Medulloblastoma	Posterior fossa	Non WNT/non SHH Group 4, Myc amp	Surgical resection (at diagnosis)	8 y ♂	NOD/SCID 4–6 weeks	Short-term cell culture in spheroids	Right cuadate/putamen (ML +2 mm, AP +0.5 mm, DV −3.3 mm)	–	44 days to onset	ATCC (www.ATCC.org)	(83)
CHLA-259	Medulloblastoma, large cell/anaplastic	Posterior fossa (4th ventricle)	ND	Surgical resection (at diagnosis)	14 y ♂	NOD/SCID 4–6 weeks	Short-term adherent cell culture	Right cuadate/putamen (ML +2 mm, AP +0.5 mm, DV −3.3 mm)	–	39–77 days	CCR (children cell line repository—www.cells.org)	(43)
DMB006	Medulloblastoma	ND	Non WNT/non SHH Group 4	Surgical resection	ND	NSG	Cell suspension from surgical specimen	Cerebellum	–		On request	(53)
DMB012	Medulloblastoma, desmoplastic	ND	SHH	ND	3 y ♀	NSG	Cell suspension from surgical specimen	Cerebellum	+	61–69 days	On request	(52)
HD-MB03	Medulloblastoma, large cell/anaplastic	4th ventricle	Non WNT/non SHH Group 3, Myc amp	Surgical resection	3 y ♂	CB17-SCID	Short-term semi-adherent cell culture	Left cerebellar hemisphere (ML−1.5 mm, AP −7 mm, DV −2 mm)	–	≤29 days (MRI)	On request	(84)
ICb-984MB	Medulloblastoma, anaplastic	Cerebellum	SHH	Surgical resection	7.8 y ♀	Rag2/SCID, 6–8 weeks	Cell suspension from surgical specimen	Right cerebellum (ML +1 mm, AP −1 mm, DV −3 mm)	–	65–93 days	On request	(16)
ICb-1078MB	Medulloblastoma, anaplastic	Cerebellum	Non WNT/non SHH Group 4	Surgical resection	11.7 y ♂	Rag2/SCID, 5–7 weeks	Cell suspension from surgical specimen	Cerebellum	–	ND	On request	(85)
ICb-1140MB	Medulloblastoma, anaplastic	Cerebellum	WNT	Surgical resection	6 y ♂	Rag2/SCID, 6–8 weeks	Cell suspension from surgical specimen	Cerebellum	–	ND	On request	(49)
ICb-1192MB	Medulloblastoma, classic	Cerebellum	WNT	Surgical resection	12.4 y ♂	Rag2/SCID, 6–8 weeks	Cell suspension from surgical specimen	Right cerebellum (ML +1 mm, AP −1 mm, DV −3 mm)	–	75–95 days	On request	(16)
ICb-1197MB	Medulloblastoma, nodular	Cerebellum	Non WNT/non SHH Group 3	Surgical resection	5 y ♂	Rag2/SCID, 6–8 weeks	Cell suspension from surgical specimen	Right cerebellum (ML +1 mm, AP −1 mm, DV −3 mm)	–	272–305 days	On request	(16)
ICb-1299MB	Medulloblastoma, anaplastic	Cerebellum	Non WNT/non SHH Group 3	Surgical resection	2.8 y ♀	Rag2/SCID, 6–8 weeks	Cell suspension from surgical specimen	Right cerebellum (ML +1 mm, AP −1 mm, DV −3 mm)	–	108–125 days	On request	(16)
ICb-1338MB	Medulloblastoma, nodular	Cerebellum	SHH	Surgical resection	0.5 y ♂	Rag2/SCID, 6–8 weeks	Cell suspension from surgical specimen	Right cerebellum (ML +1 mm, AP −1 mm, DV −3 mm)	–	140–203 days	On request	(16)
ICb-1487MB	Medulloblastoma, classic	Cerebellum	Non WNT/non SHH Group 4	Surgical resection	6.9 y ♂	Rag2/SCID, 5–7 weeks	Cell suspension from surgical specimen	Cerebellum	–	ND	On request	(85)
ICb-1494MB	Medulloblastoma, anaplastic	Cerebellum	Non WNT/non SHH Group 3	Surgical resection	5.2 y ♀	Rag2/SCID, 6–8 weeks	Cell suspension from surgical specimen	Right cerebellum (ML +1 mm, AP −1 mm, DV −3 mm)	–	55–105 days	On request	(16)
ICb-1572MB	Medulloblastoma, large cell	Cerebellum	Non WNT/non SHH Group 3	Surgical resection	14.8 y ♂	Rag2/SCID, 6–8 weeks	Cell suspension from surgical specimen	Right cerebellum (ML +1 mm, AP −1 mm, DV −3 mm)	–	40–82 days	On request	(16)
ICb-1595MB	Medulloblastoma, anaplastic	Cerebellum	Non WNT/non SHH Group 3	Surgical resection	1.2 y ♂	Rag2/SCID, 5–7 weeks	Cell suspension from surgical specimen	Cerebellum	–	ND	On request	(85)
ICb-Z61109MB	Medulloblastoma, anaplastic	Cerebellum	ND	Surgical resection	7 y ♂	Rag2/SCID, 6–8 weeks	Cell suspension from surgical specimen	Right cerebellum (ML +1 mm, AP −1 mm, DV −3 mm)	–	ND	On request	(68)
ICb-J1017MB	Medulloblastoma, anaplastic	Cerebellum	Non WNT/non SHH Group 3	Surgical resection	9 y ♂	Rag2/SCID, 6–8 weeks	Cell suspension from surgical specimen	Right cerebellum (ML +1 mm, AP −1 mm, DV −3 mm)	–	ND	On request	(68)
MB3W1	Medulloblastoma, anaplastic	4th ventricle	Non WNT/non SHH Group 3, Myc amplification	Surgical resection	1.8 y ♂	NOD/SCID, 10–13 weeks	Short-term cell culture in spheroids	Right cerebellum	+	28–55 days	On request	(86)
MB-LU-181	Medulloblastoma	ND	Non WNT/non SHH Group 3	Surgical resection	4 y ♂	NOD/SCID, 8 weeks	Short-term cell culture in spheroids	Right cerebellum (ML +1 mm, AP −2 mm, DV −2.5 mm)	–	70–126 days	On request	(87)
Med-113FH	Medulloblastoma, large cell/anaplastic	Cerebellum	SHH	Surgical resection	9.9 y ♂	NSG	Cell suspension from surgical specimen	Right cerebellum (ML +2 mm, AP −2 mm, DV −2 mm)	–	72–112 days	BTRL (Brain Tumor Resource Lab—https://research.fhcrc.org)	(72)
Med-114FH	Medulloblastoma, large cell/anaplastic	Cerebellum	Non WNT/non SHH Group 3, Myc amplification	Surgical resection	6.6 y ♀	NSG	Cell suspension from surgical specimen	Right cerebellum (ML +2 mm, AP −2 mm, DV −2 mm)	–	31–60 days	BTRL (Brain Tumor Resource Lab—https://research.fhcrc.org)	(72)
Med-1512FH	Medulloblastoma, desmoplastic	Cerebellum	Non WNT/non SHH Group 4	Surgical resection	6 y ♀	NSG	Cell suspension from surgical specimen	Right cerebellum (ML +2 mm, AP −2 mm, DV −2 mm)	–	124–226 days	BTRL (Brain Tumor Resource Lab—https://research.fhcrc.org)	(72)
Med-1712FH	Medulloblastoma, desmoplastic	Cerebellum	SHH	Surgical resection	4.9 y ♂	NSG, 6–10 week	Cell suspension from surgical specimen	Right cerebellum (ML +2 mm, AP −2 mm, DV −2 mm)	–	86–157 days	BTRL (Brain Tumor Resource Lab—https://research.fhcrc.org)	(53)
Med-1911FH	Medulloblastoma, large cell/anaplastic	Cerebellum	Non WNT/non SHH Group 3, Myc amplification	Surgical resection	3.5 y ♀	NSG	Cell suspension from surgical specimen	Right cerebellum (ML +2 mm, AP −2 mm, DV −2 mm)	–	55–128 days	BTRL (Brain Tumor Resource Lab—https://research.fhcrc.org)	(72)
Med-210FH	Medulloblastoma (with myogenic differentiation)	Cerebellum	Non WNT/non SHH Group 3	Surgical resection	5.2 y ♀	NSG	Cell suspension from surgical specimen	Right cerebellum (ML +2 mm, AP −2 mm, DV −2 mm)	–	18–224 days	BTRL (Brain Tumor Resource Lab—https://research.fhcrc.org)	(72)
Med-211FH	Medulloblastoma, classic	Cerebellum	Non WNT/non SHH Group 3, Myc amplification	Surgical resection	2.8 y ♂	NSG 6–8 weeks	Cell suspension from surgical specimen (serial transplantation)	Right cerebellum (ML +2 mm, AP −2 mm, DV −3 mm)	–	42–64 days	BTRL (Brain Tumor Resource Lab—https://research.fhcrc.org)	(51)
Med-2112FH	Medulloblastoma, large cell/anaplastic	Cerebellum	Non WNT/non SHH Group 3, Myc amplification	Surgical resection	7 y ♂	NSG	Cell suspension from surgical specimen	Right cerebellum (ML +2 mm, AP −2 mm, DV −2 mm)	–	52–91 days	BTRL (Brain Tumor Resource Lab—https://research.fhcrc.org)	(72)
Med-2312FH	Medulloblastoma, classic	Cerebellum	Non WNT/non SHH Group 4	Surgical resection	2.8 y ♀	NSG	Cell suspension from surgical specimen	Right cerebellum (ML +2 mm, AP −2 mm, DV −2 mm)	–	105–153 days	BTRL (Brain Tumor Resource Lab—https://research.fhcrc.org)	(72)
Med-314FH	Medulloblastoma, classic	Cerebellum	SHH	Surgical resection (recurrence)	10 y ♀	NSG	Cell suspension from surgical specimen	Right cerebellum (ML +2 mm, AP −2 mm, DV −2 mm)	–	56–77 days	BTRL (Brain Tumor Resource Lab—https://research.fhcrc.org)	(72)
Med-411FH	Medulloblastoma, large cell/anaplastic	Cerebellum	Non WNT/non SHH Group 3	Surgical resection	3 y ♂	NSG, 6–10 week	Cell suspension from surgical specimen	Right cerebellum (ML +2 mm, AP −2 mm, DV −2 mm)	+	29–39 days	BTRL (Brain Tumor Resource Lab—https://research.fhcrc.org)	(53)
Med-511FH	Medulloblastoma	Cerebellum	Non WNT/non SHH Group 3, Myc amplification	Surgical resection (primary tumor)	ND	CD1 nu/nu	Cell suspension from surgical specimen	Cortex	+	62–68 days	on request (Dr. Olson, Fred Hutch)	(54)
Med-610FH	Medulloblastoma, large cell/anaplastic	Cerebellum	Non WNT/non SHH Group 4	Surgical resection	5.3 y ♂	NSG	Cell suspension from surgical specimen	Right cerebellum (ML +2 mm, AP −2 mm, DV −2 mm)	–	148–187 days	BTRL (Brain Tumor Resource Lab—https://research.fhcrc.org)	(72)
Med-813FH	Medulloblastoma, classic	Cerebellum	SHH	Surgical resection	2.6 y ♂	NSG	Cell suspension from surgical specimen	Right cerebellum (ML +2 mm, AP −2 mm, DV −2 mm)	–	32–78 days	BTRL (Brain Tumor Resource Lab—https://research.fhcrc.org)	(72)
Med-913FH	Medulloblastoma, classic	Cerebellum	WNT	Surgical resection	7.5 y ♀	NSG	Cell suspension from surgical specimen	Right cerebellum (ML +2 mm, AP −2 mm, DV −2 mm)	–	175–415 days	BTRL (Brain Tumor Resource Lab—https://research.fhcrc.org)	(72)
nMED1	Medulloblastoma, NOS	Cerebellum	ND	Surgical resection	3.4 y	ND	Short-term adherent cell culture	Right cerebral hemisphere (ML +2 mm, AP +2 mm)	–	ND	Children's Brain Tumour Research Centre, Nottingham	(38)
nMED2	Medulloblastoma, NOS	Frontal bilateral (metastasis)	ND	Surgical resection (recurrence)	10.6 y	ND	Short-term adherent cell culture	Right cerebral hemisphere (ML +2 mm, AP +2 mm)	–	ND	Children's Brain Tumour Research Centre, Nottingham	(38)
PBT-07FH	Medulloblastoma	ND	Non WNT/non SHH Group 3	Surgical resection	3.5 y ♀	NSG	Cell suspension from surgical specimen	Right cerebral hemisphere (ML +1 mm, AP +1.5 mm, DV −2 mm)	–	67–169 days	BTRL (Brain Tumor Resource Lab—https://research.fhcrc.org)	(72)
RCMB18	Medulloblastoma, anaplastic	ND	SHH	Surgical resection	7 y ♂	NSG 6–8 weeks	Cell suspension from surgical specimen	Cerebellum	+	34–58 days	on request (Dr. Wechsler-Reya, Sanford-Burnham medical Discovery institute)	(52)
RCMB28	Medulloblastoma	ND	Non WNT/non SHH Group 3	ND	ND	NSG 6–86–8 weeks	Cell suspension from surgical specimen	Cerebellum	–	ND	On request	(53)
RCMB32	Medulloblastoma	ND	SHH	ND	ND	NSG 6–8 weeks	Cell suspension from surgical specimen	Cerebellum	–	ND	On request	(53)
SU-MB-02	Medulloblastoma, large cell/anaplastic	ND	Non WNT/non SHH Group 3, Myc amplification	Autopsy (leptomeningial spread)	3 y ♂	NSG 4–6 weeks	Short-term cell culture in spheroids	Cerebellum (AP −2 mm, DV −2 mm)	+	33–40 days	On request (Dr. Cho, Stanford)	(65)
SU-MB-09	Medulloblastoma	ND	Non WNT/non SHH Group 4	Surgical resection	9 y ♀	NSG 4–6 weeks	Short-term cell culture in spheroids	Cerebellum (AP −2 mm, DV −2 mm)	+	83–100 days	On request (Dr. Cho, Stanford)	(65)
UM-MB1	Medulloblastoma, NOS	Posterior fossa	ND	Surgical resection	4 y ♀	CD1 nu/nu, 4 weeks	Short-term adherent cell culture	Right cerebral hemisphere (ML +1 mm, AP +2 mm, DV −3.5 mm)	–	ND	On request	(44)
**EMBRYONAL TUMORS—OTHER**												
BT183	Embryonal tumor with multilayered rosettes, C19MC-altered	ND	C19MC amplification	ND	2 y ♂	NOD/SCID, 6–8 weeks	Short-term cell culture in spheroids	Right striatum (ML +2 mm, AP −1 mm, DV −3 mm)	+	8–45 days	On request	(88)
IC-2664 PNET	CNS embryonal tumor, NOS	ND	ND	Surgical resection	14 y ♀	Rag2/SCID, 6–8 weeks	Cell suspension from surgical specimen	Right cerebral hemisphere (ML +1 mm, AP +1.5 mm, DV −3 mm)	–	48–76 days	On request	(89)
NCH3602	Embryonal tumor with multilayered rosettes, C19MC-altered	Right hemisphere	C19MC amplification	Surgical resection (at diagnosis)	2 y	NSG, 6–8 weeks	Short-term cell culture in spheroids	Right striatum (ML +2,5 mm, AP −1 mm, DV −3 mm)	+	ND	On request	(90)
ncPNET	CNS embryonal tumor, NOS	Cerebrum (left frontal)	ND	Surgical resection	5 y	ND	Short-term adherent cell culture	Right cerebral hemisphere (ML +2 mm, AP +2 mm)	–	ND	Children's Brain Tumour Research Centre, Nottingham	(38)
ATRT-310FH	Atypical teratoid/rhabdoid tumor	Anterior cranial fossa	ATRT SHH	Surgical resection	6.1 y ♀	NSG, 6–8 weeks	Cell suspension from surgical specimen (serial transplantation)	Right cerebral hemisphere (ML +1 mm, AP +1.5 mm, DV −2 mm)	–	33–143 days	BTRL (Brain Tumor Resource Lab—https://research.fhcrc.org)	(51)
ATRT-312FH	Atypical teratoid/rhabdoid tumor	Cortex (parietal lobe)	ATRT MYC	ND	1.8 y ♂	NSG	Cell suspension from surgical specimen	Right cerebral hemisphere (ML +1 mm, AP +1.5 mm, DV −2 mm)	–	40–89 days	BTRL (Brain Tumor Resource Lab—https://research.fhcrc.org)	(72)
CHLA-06-ATRT	Atypical teratoid/rhabdoid tumor	Posterior fossa	INI-1 loss	Surgical resection (primary tumor)	3 mo ♀	ND	Short-term semi-adherent cell culture	Right striatum (ML +2 mm, AP −3 mm, DV −3 mm)	–	14–20 days	ATCC (www.ATCC.org/) CCR (childhood cancer repository—www.cccells.org)	(55)
CHLA-266	Atypical teratoid/rhabdoid tumor	posterior fossa	INI-1 loss	Surgical resection (at diagnosis)	2.5 y ♀	NSG 6–8 weeks	Short-term adherent cell culture	Right cuadate/putamen (ML +2 mm, AP +0.5 mm, DV −3.3 mm)	–	40–50 days	CCR (childhood cancer repository—www.cccells.org)	(43)
SU-ATRT-02	Atypical teratoid/rhabdoid tumor	Supratentorial	ND	Surgical resection (primary tumor)	2 y ♂	NSG 5–6 weeks	Short-term cell culture in spheroids	Right striatum (ML +2 mm, AP −2 mm, DV −3.5 mm)	+	50–63 days	On request	(65)
**GERM CELL TUMORS**												
IC-6999GCT	Germinoma	C6 spinal cord	ND	Surgical resection (metastasis-recurrence)	16 y ♂	Rag2/SCID, 6–8 weeks	Cell suspension from surgical specimen	Right cerebral hemisphere (ML +1 mm, AP +1.5 mm, DV −3 mm)	–	80–242 days	On request	(62)
IC-9320GCT	Germinoma	Supratentorial	KIT D816H	Surgical resection (metastasis)	1.5 y ♀	Rag2/SCID, 6–8 weeks	Cell suspension from surgical specimen	Right cerebral hemisphere (ML +1 mm, AP +1.5 mm, DV −3 mm)	–	60–160 days	On request	(62)
**TUMORS OF THE SELLAR REGION**												
adaCP 1	Adamantinomatous craniopharyngeoma	ND	CTNNB1 mutation	Surgical resection	16 y ♀	NSG, 5–8 weeks	Tumor tissue	Right cerebral hemisphere (ML +3 mm)	–	ND	On request	(33)
ACP1	Adamantinomatous craniopharyngeoma	Sellar region	CTNNB1 mutation	Surgical resection	9 y ♂	NMRI nu/nu, 5 weeks	Tumor tissue	Right cerebral hemisphere (ML +3 mm)	–	ND	On request	(34)

*Indicated are the location, classification, and moment of collection of the original tumor sample, patient characteristics, mouse/rat strain used, tumor preparation, and injection site. References concern the first manuscripts describing the model only. To facilitate the choice of appropriate models for the preclinical therapeutic studies, this table also indicates whether the model allows for bioluminescence imaging (BLI), time to tumor growth/euthanasia (as estimated from Kaplan-Meijer curves, unless otherwise indicated), and source where to obtain cells. “On request” refers to the corresponding author of the reference. FL, Fluorescence (MION-Rh); ND, Not described*.
